# An Integrated Expression Profiling Reveals Target Genes of TGF-β and TNF-α Possibly Mediated by MicroRNAs in Lung Cancer Cells

**DOI:** 10.1371/journal.pone.0056587

**Published:** 2013-02-20

**Authors:** Akira Saito, Hiroshi I. Suzuki, Masafumi Horie, Mitsuhiro Ohshima, Yasuyuki Morishita, Yoshimitsu Abiko, Takahide Nagase

**Affiliations:** 1 Department of Respiratory Medicine, Graduate School of Medicine, The University of Tokyo, Bunkyo-ku, Tokyo, Japan; 2 Division for Health Service Promotion, The University of Tokyo, Bunkyo-ku, Tokyo, Japan; 3 Department of Molecular Pathology, Graduate School of Medicine, The University of Tokyo, Bunkyo-ku, Tokyo, Japan; 4 Department of Biochemistry, Ohu University School of Pharmaceutical Sciences, Tomitamachi, Koriyama, Fukushima, Japan; 5 Department of Biochemistry and Molecular Biology, Nihon University School of Dentistry at Matsudo, Matsudo, Chiba, Japan; Cincinnati Children’s Hospital Medical Center, United States of America

## Abstract

EMT (epithelial-mesenchymal transition) is crucial for cancer cells to acquire invasive phenotypes. In A549 lung adenocarcinoma cells, TGF-β elicited EMT in Smad-dependent manner and TNF-α accelerated this process, as confirmed by cell morphology, expression of EMT markers, capacity of gelatin lysis and cell invasion. TNF-α stimulated the phosphorylation of Smad2 linker region, and this effect was attenuated by inhibiting MEK or JNK pathway. Comprehensive expression analysis unraveled genes differentially regulated by TGF-β and TNF-α, such as cytokines, chemokines, growth factors and ECM (extracellular matrices), suggesting the drastic change in autocrine/paracrine signals as well as cell-to-ECM interactions. Integrated analysis of microRNA signature enabled us to identify a subset of genes, potentially regulated by microRNAs. Among them, we confirmed TGF-β-mediated induction of miR-23a in lung epithelial cell lines, target genes of which were further identified by gene expression profiling. Combined with in silico approaches, we determined HMGN2 as a downstream target of miR-23a. These findings provide a line of evidence that the effects of TGF-β and TNF-α were partially mediated by microRNAs, and shed light on the complexity of molecular events elicited by TGF-β and TNF-α.

## Introduction

Lung cancer is the most frequent cancer type, which causes death of more than one million people every year. Understanding of molecular events which govern invasive/metastatic spread of cancer cells is crucial for developing novel therapeutics of lung cancer. Epithelial-mesenchymal transition (EMT) is the differentiation switch directing epithelial cells to acquire mesenchymal phenotypes, which plays key roles during embryonic development as well as cancer invasion/metastasis. The hallmark of EMT is E-cadherin downregulation and subsequent loss of cell-cell adhesions, which is coupled with increased expression of mesenchymal markers including N-cadherin and vimentin. Additionally EMT is accompanied with cell morphological changes from ‘cuboidal’ to ‘spindle-like’ appearances, which correspond to actin reorganization and cytoskeltal alterations, leading to acquisition of the fibroblast-like migratory phenotype [1], [2].

Transforming growth factor (TGF)-β plays a central role in the regulation of EMT and exhibits its pleiotropic effects through binding to receptors type I (TβR-I) and type II (TβR-II). Upon ligand-induced heteromeric complex formation between TβR-I and TβR-II, TβR-I is phosphorylated by TβR-II and mediates specific intracellular signaling through phosphorylation of receptor-regulated Smads (R-Smads: Smad2 and Smad3 for TGF-β). Phosphorylated R-Smads interact with Smad4 and translocate into the nucleus, where they regulate transcription of target genes [3], [4]. TGF-β is often overexpressed in tumor tissues, and facilitates cancer progression through a diverse repertoire of tumor-cell-autonomous and host–tumor interactions, including enhancement of cell motility and invasion, which involves the process of EMT [5].

Accumulating evidence unravels the molecular mechanisms by which inflammatory responses promote tumor progression [6]. Tumor necrosis factor (TNF)-α is one of the most potent pro-inflammatory cytokines produced in the tumor microenvironment. Upon stimulation, activated IKK (IκB kinase) phosphorylates NFκB inhibitor (IκB) and triggers its rapid degradation through proteasome proteolysis, resulting in the liberation of NFκB, which then translocates to the nucleus and induces a myriad of gene expression involved in immune response [7]. The contribution of NFκB signaling to the initiation and progression of cancer is clearly documented, and several lines of evidence demonstrate that TNF-α and/or NFκB signaling plays a key role in the regulation of EMT [8], [9], [10].

Noncoding microRNAs (miRNAs) attract increasing attention as key components of cell signaling, which regulate expression levels of multiple proteins, primarily by binding to the 3′ untranslated region (UTR) of targets. Important roles for miRNAs have been shown in tumor progression by modulation of cell differentiation, proliferation, invasion, and metastasis. MicroRNA-200 (miR-200) and miR-205 are critically involved in maintaining the epithelial cell phenotype and are suppressed by TGF-β [11]. It is also reported that miR-21 and miR-31 are synergically induced by TGF-β and TNF-α, which facilitate cancer cell invasion [12].

Recent studies have shown that TNF-α enhances TGF-β-mediated EMT in lung cancer/epithelial cells [13], [14], [15], suggesting the potential crosstalks between these signals. However, little is known about the molecular events how these signals are orchestrated to modulate EMT. We have previously demonstrated that TGF-β induces EMT in A549 lung adenocarcinoma cells [16], which harbor an activating K-ras mutation and form a tumor with well-differentiated adenocarcinoma histology when subcutaneously injected into immunocompromized mice [17], [18]. In the present study, we explored the underlying mechanisms of EMT mediated by TGF-β and TNF-α in A549 cells. In search of the target genes and miRNAs, we performed comprehensive expression analyses in combination with in silico screening. These data delineated subsets of genes differentially or cooperatively regulated by TGF-β and TNF-α, and identified miR-23a as a miRNA target of TGF-β. These analyses further implied the possibility that a subset of TGF-β target genes could be regulated by miRNAs, shedding light on the complexity of molecular events elicited by TGF-β and TNF-α in lung cancer cells.

## Materials and Methods

### Reagents and Antibodies

TGF-β1 and TNF-α were purchased from Sigma-Aldrich (St. Louis, MO) and R&D Systems (Minneapolis, MN), and were used at the concentration of 5 ng/ml and 10 ng/ml, respectively. Anti-Smad2, phosphorylated (phospho-) Smad2 at Ser 245/250/255, phospho-Smad2 at Ser 465/467, Smad3, phospho-Smad3 at Ser 423/425, Smad4, Erk, phospho-Erk, p38, phospho-p38, phospho-c-Jun and E-cadherin antibodies were from Cell Signaling (Beverly, MA). Anti-N-cadherin antibody was from BD Pharmingen (Transduction Laboratories, Lexington, KY). Anti-α-tubulin antibody was from Sigma-Aldrich. Anti-HMGN2 antibody was from Millipore (Darmstadt, Germany). LY-364947 (TβR-I inhibitor) was used at the concentration of 3 µM. U0126 (MEK 1/2 inhibitor), SP600125 (JNK inhibitor) and SB203580 (p38 inhibitor) were used at the concentration of 25 µM.

### Cell Culture

A549 lung adenocarcinoma cells [19] were gifted from Cell Resource Center for Biomedical Research, Institute of Development, Aging and Cancer, Tohoku University (Sendai, Japan). NCI-H441 (H441) lung adenocarcinoma cells and HEK293T cells were from American Type Culture Collection. Transformed human bronchial epithelial cells (BEAS2B cells) were purchased from Summit Pharmaceuticals International (Tokyo, Japan). Cells were photographed using a phase-contrast microscope (Olympus, Tokyo, Japan). Cell circularity was measured by NIH Image J software.

### Transfection

Lipofectamine RNAiMAX reagent (Invitrogen) was used for siRNA transfection into A549 cells, and final concentration of siRNA was 20 nM. Human Smad4 siRNA (Stealth RNAi VHS41118) and negative control siRNA were purchased from Invitrogen. The transfected cells were cultured for 48 h and seeded at the same cell density, followed by incubation with TGF-β1 and/or TNF-α. To assess the effect of miR-23a, 10 nM of synthetic precursor miR-23a (pre-miR-23a) or Cy3-labelled negative control (Applied Biosystems, Carlsbad, CA) was transfected into A549 cells using Lipofectamine RNAiMAX reagent. The transfection efficiency judged by Cy3 fluorescence was more than 95% as confirmed by flow cytometry. For microarray analysis, RNA sample was collected 48 h after transfection.

### Immunoblot Analysis

Cells were put on ice and rinsed with PBS, then lysed in lysis buffer (20 mM Tris-HCl, pH 7.5, 150 mM NaCl, 1 mM EDTA, 0.5% Nonidet P-40) supplemented with protease and phosphatase inhibitors for immunoblotting. Following centrifugation at 15000 rpm for 15 min, cell lysates were quantitated for protein content by BCA Protein Assay Kit (Pierce, Rockford, IL) and equal amounts of total proteins were processed to SDS-PAGE, followed by semi-dry transfer of the proteins to nitrocellulose membrane. Non-specific binding of proteins to the membrane was blocked by incubation with Amersham ECL Prime Blocking Agent (GE Healthcare, Buckinghamshire, UK) in TBS-T buffer (50 mM Tris-HCl, pH 7.4, 150 mM NaCl, 0.05% Tween-20). The immunoblotted proteins were detected with the ECL blotting system and LightCapture/Ez-Capture imaging system (ATTO, Tokyo, Japan).

### RNA Isolation and RT-PCR

Total RNA was isolated using the RNeasy Mini Kit (Qiagen, Hilden, Germany). The synthesis of cDNA was performed using SuperScript III First-Strand Synthesis System (Invitrogen, Carlsbad, CA), according to the manufacturer’s instructions. Quantitative RT-PCR analysis was performed using Mx-3000P (Stratagene, La Jolla, CA) and QuantiTect SYBR Green PCR (Qiagen). Expression level was normalized to that of glyceraldehyde-3-phosphate dehydrogenase (*GAPDH*). Primer sequences are shown in Table S1. MicroRNA was isolated using the miRNeasy Mini Kit (Qiagen). Mature miR-23a was reverse-transcribed, and quantitative PCR was performed using TaqMan microRNA assays (Applied Biosystems). Expression level was normalized to that of U6.

### Gelatin Zymography

Conditioned media without FBS were collected and equal amounts of protein were mixed with 4 × non-reducing SDS-PAGE sample buffer. The samples were applied to a 10% (w/v) polyacrylamide gel impregnated with 1 mg/ml gelatin (Sigma-Aldrich). After electrophoresis, SDS was removed from the gel by washing 3 times for 20 min in 2.5% Triton X-100 solution. Then the gels were incubated overnight with gentle shaking at 37°C in buffer (50 mM Tris-HCl, pH 7.6, 5 mM CaCl_2_, 200 mM NaCl, 0.02% Brij35). The gel was stained with 0.5% Coomassie blue R250 in 50% methanol and 5% acetic acid for 2 h at room temperature, and subsequently destained with 40% methanol-10% acetic acid solution until the bands became clear.

### Invasion Assay

Cell invasion assay was performed using cell culture Inserts with 8 µm pore size (BD Biosciences, Franklin Lakes, NJ). The upper surface of the chamber was coated with growth factor reduced Matrigel (BD Biosciences). A549 cells (4×10^5^ cells/well) resuspended in serum free media were seeded in the upper side of the chamber. In the lower side of the chamber, the growth medium supplemented with 10% FBS was added. TGF-β1 and/or TNF-α were added into both sides of the chamber. After 24 h, cells on the lower surface of the chamber were trypsinized, resuspended in PBS and counted with a hemocytometer. The experiments were performed with triplicate, and repeated 3 times. The data are presented as the mean of the ratio compared to control, out of 3 independent experiments.

### Expression Profiling

Gene expression profiling was performed using a GeneChip® Human Gene 1.0 ST Array (Affymetrix, Santa Clara, CA). The microarray processing was carried out according to the manufacturer’s instructions. The expression of 19,734 genes was monitored, and the data was imported into GeneSpring GX software (Agilent Technologies, Santa Clara, CA) for the selection of induced and repressed genes.

The expression of 1223 mature microRNAs was profiled using Exiqon’s miRCURY LNA Array, 6th generation (Filgen, Nagoya, Japan). Briefly, RNA samples were checked for RNA integrity on Bioanalyzer 2100 (Agilent Technologies, Wilmington, DE), labeled with Hy3, and hybridized. Slides were scanned using GenePix®4000B (Molecular Devices, Union City, CA), and the images were digitized with Array-Pro Analyzer Ver. 4.5 (Media Cybernetic, Silver Spring, MD). Finally, data were normalized and expressed as fold increase with the MicroArray Data Analysis Tool Ver. 3.2 (Filgen).

Ingenuity Pathways Analysis (IPA) (Ingenuity Systems, Mountain View, CA) was used for the mapping of gene expression data into relevant pathways based on the gene’s functional annotation and known molecular interactions. For integrated analysis of miRNA and mRNA signatures, the miRNA Target Filter in IPA was employed, which extracted possible miRNA-mRNA interactions based on the databases such as TarBase, miRecords and TargetScan.

### Luciferase Reporter Assay

Pri-miR23a expression vector was generated by cloning the short fragment of pri-miRNA containing pre-miRNA and flanking sequence into pcDNA6.2-GW/EmGFP-miR (Invitrogen). For the reporter construct, the 3′UTR segment of human HMGN2 gene was cloned into the luciferase reporter vector. The primer sequences used are given in Table S2. HEK293T cells were transfected with each reporter construct with or without pri-miR23a expression vector using FuGENE6 (Roche, Basel, Switzerland). The ratio of renilla to firefly luciferase was measured using the Dual-Luciferase Reporter Assay System (Promega, Madison, WI).

## Results

### TNF-α Enhances TGF-β-mediated EMT in A549 Lung Cancer Cells

**Figure 1:**
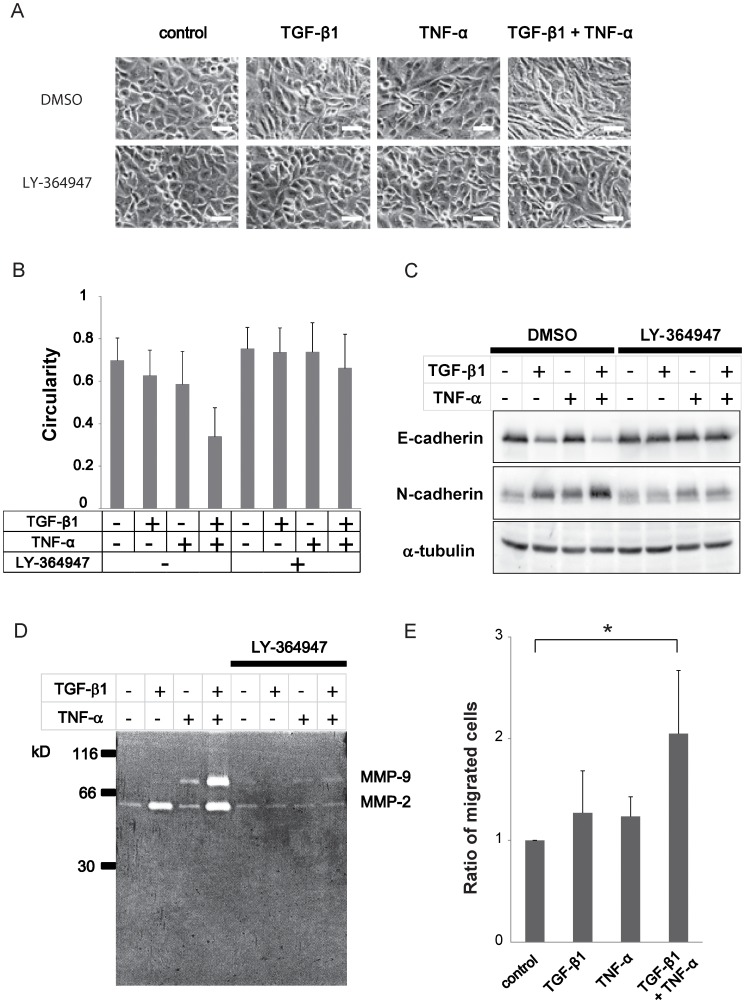
TNF-α enhances TGF-β-mediated EMT in A549 lung cancer cells. (A) A549 cells were pretreated with LY-364947 (TβR-I inhibitor) or control DMSO for 60 min, further cultured with 5 ng/ml TGF-β1 and/or 10 ng/ml TNF-α for 48 h, and analyzed by phase-contrast microscopy. Bar: 50 µm. (B) Cell circularity was measured using Image J software to quantify cell morphological change following the described treatment. (C) Immunoblotting analyses of E-cadherin and N-cadherin in A549 cells stimulated with TGF-β1 and/or TNF-α for 48 h in the presence or absence of LY-364947. α-tubulin was used as loading control. (D) Gelatin zymography. A549 cells treated as described were cultured with serum free media for additional 48 h. The conditioned media were collected and the same amount of protein was electrophoresed. Gelatin digestion by activated MMP-2 and MMP-9 was visualized by Coomassie blue staining. (E) Cell invasion assay. The migrated cells through the culture inserts coated with Matrigel were trypsinized and counted. Each experiment was performed in triplicate and the averaged relative ratios from 3 independent experiments were presented. Error bars: SD. *P<0.05 (Student’s t-test).

First we characterized the effect of TGF-β and/or TNF-α on EMT in A549 lung cancer cells. At confluency, A549 cells displayed cobblestone-like appearances and TGF-β treatment led to cell morphological change to elongated shape. TNF-α was also potent in inducing cell morphological change to spindle-like appearances. As previously reported, costimulation of TGF-β and TNF-α resulted in dramatic change of cell shape to fibroblast-like appearances [14], which was clearly distinguishable from those observed in the cells treated with TGF-β or TNF-α alone (Figure 1A, upper panels).

Next we examined the effect of the TβR-I kinase inhibitor, LY-364947. Blockade of endogenous TGF-β signaling by LY-364947 resulted in uniformly cuboidal cell morphology, and the effect of exogenous TGF-β was clearly abrogated in the presence of LY-364947. On the other hand, TNF-α-mediated cell morphological change was still observed, albeit to a lesser extent, in the cells pretreated with LY-364947, indicative of the effect of TNF-α alone exerted in the absence of endogenous TGF-β signaling (Figure 1A, lower panels).

These morphological changes were quantified by measuring the cell circularity (Figure 1B). In the cells costimulated with TGF-β and TNF-α, cell circularity was markedly reduced, suggesting the synergic effect on cell morphology. In the presence of LY-364947, their effects were mostly inhibited, implying the critical contribution of TGF-β to the observed synergic effect.

The process of EMT is accompanied by downregulation of E-cadherin and upregulation of N-cadherin, which is termed as cadherin switch [1], [2]. In the following experiments, we examined the expression of E-cadherin and N-cadherin, as epithelial and mesenchymal markers, respectively. E-cadherin expression was mostly abrogated by TGF-β treatment whereas TNF-α alone displayed a marginal effect on E-cadherin downregulation at protein level (Figure 1C). Consistent with the change in E-cadherin expression, N-cadherin was upregulated by treatment with TGF-β or TNF-α. Furthermore, in accordance with the dramatic change in cell morphology, TNF-α enhanced the effect of TGF-β on epithelial/mesenchymal markers. The effect of TGF-β on the expression of E-cadherin/N-cadherin was inhibited by LY-364947 whereas TNF-α-mediated upregulation of N-cadherin was also observed regardless of LY-364947 treatment.

EMT is accompanied with enhancement of protease activities that facilitate degradation of basement membrane and extracellular matrices (ECM) surrounding tumor cells, which is critical for tumor invasion/metastasis. To analyze proteolytic activities of matrix metalloproteinases (MMPs) in the cells treated with TGF-β and/or TNF-α, we performed gelatin zymography (Figure 1D). TGF-β enhanced the activity of MMP-2 while TNF-α enhanced that of MMP-9. Notably, costimulation with TGF-β and TNF-α drastically promoted the activities of both MMP-2 and MMP-9, suggesting the synergic effect to enhance MMP activities. In the presence of LY-364947, the effect of TGF-β was clearly inhibited, whereas the effect of TNF-α to enhance MMP-9 activity was still observed albeit to a lesser extent, in the absence of endogenous TGF-β signaling.

To examine the functional aspect of EMT, we performed invasion assay, which utilizes chambers coated with Matrigel, mimicking the basement membrane. TGF-β or TNF-α treatment resulted in modestly increased number of invading cells on the lower face of the chambers, whereas costimulation with TGF-β and TNF-α led to enhanced invasive capacity, in agreement with the above-observed changes (Figure 1E). TGF-β treatment failed to enhance invasive capacity as robust as the changes in EMT markers, suggesting that changes in markers are not directly linked to cell invasiveness. The process of invasion includes enhanced cell motility and proteolytic activities. Together with the results of gelatin zymography, increased invasive capacity in the cells costimulated with TGF-β and TNF-α appeared to be related to enhanced MMP activities.

Taken together, TGF-β-mediated EMT was clearly enhanced by TNF-α as judged by cell morphology, EMT markers, gelatin lysis and cell invasion. TNF-α alone could also induce part of these changes even in the presence of LY-364947, such as N-cadherin upregulation and MMP-9 activation. These observations prompted us to explore the possible crosstalks between TGF-β and TNF-α, and molecular events which regulate EMT in A549 cells.

### TGF-β-mediated EMT is Smad-dependent

Smads are the major transducer of TGF-β signaling; Smad2 and Smad3 are phosphorylated by TβR-I, and form complexes with Smad4. These complexes accumulate in the nucleus and regulate transcription of target genes [20]. Besides Smad-mediated transcription, TGF-β activates other signaling cascades, including MAPK (mitogen-activated protein kinase) pathways [21].

**Figure 2:**
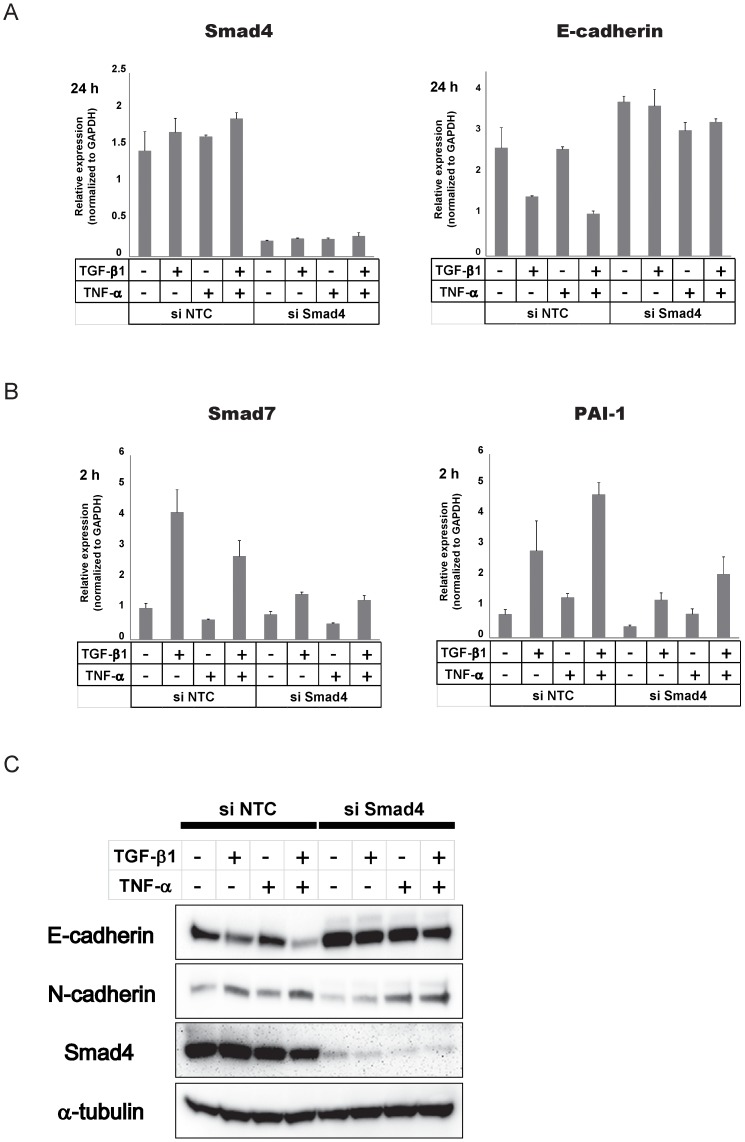
TGF-β-mediated EMT is Smad-dependent. (A–B) A549 cells were transfected with siRNAs for Smad4 (si Smad4), or negative control siRNAs (si NTC) and cultured for 48 h. The cells were further cultured with 5 ng/ml TGF-β1 and/or 10 ng/ml TNF-α for 2 h (B) or 24 h (A), and RNA was collected. Quantitative PCR was performed for Smad4, E-cadherin, Smad7 and PAI-1 at the indicated time. Expression was normalized to that of GAPDH. Error bars: SD. (C) Cell lysates were collected 48 h after TGF-β1 and/or TNF-α treatment. Immunoblotting was performed for E-cadherin, N-cadherin and Smad4. α-tubulin was used as loading control.

To examine whether Smad-mediated signaling is involved in the regulation of EMT in A549 cells, we knocked down endogenous Smad4, which is commonly required for the Smad-mediated transcriptional regulation. Transfection of siRNA effectively silenced Smad4 expression (Figure 2A, left), and further suppressed the expression of Smad-regulated target genes of TGF-β, such as Smad7 and PAI-1 (plasminogen activator inhibitor-1, also known as *SERPINE1*) (Figure 2B). In this setting, TGF-β failed to downregulate E-cadherin as judged by quantitative RT-PCR (Figure 2A, right), suggesting that TGF-β-mediated EMT is mainly regulated by Smad pathway. These effects were further confirmed by immunoblotting. TGF-β failed to downregulate E-cadherin or upregulate N-cadherin efficiently as control siRNA transfected cells when endogenous Smad4 was silenced (Figure 2C).

### TNF-α Phosphorylates Smad2 Linker Region

R-Smad and Smad4 contain conserved N-terminal MH1 and C-terminal MH2 domains, flanking the linker segment. Ligand-induced interaction of R-Smads with activated TβR-I results in direct phosphorylation of C-terminal SSXS motif [20], which is the key event of Smad activation. Moreover, other kinase pathways further regulate Smad signaling via phosphorylation of the linker region of R-Smads [21], [22]. Besides NFκB signaling, TNF-α is known to elicit MAPK pathways, which are consisted of three subfamilies, i.e. extracellular signal-regulated kinase (Erk) 1 and 2, p38 MAPK and the c-Jun N-terminal kinase (JNK).

**Figure 3:**
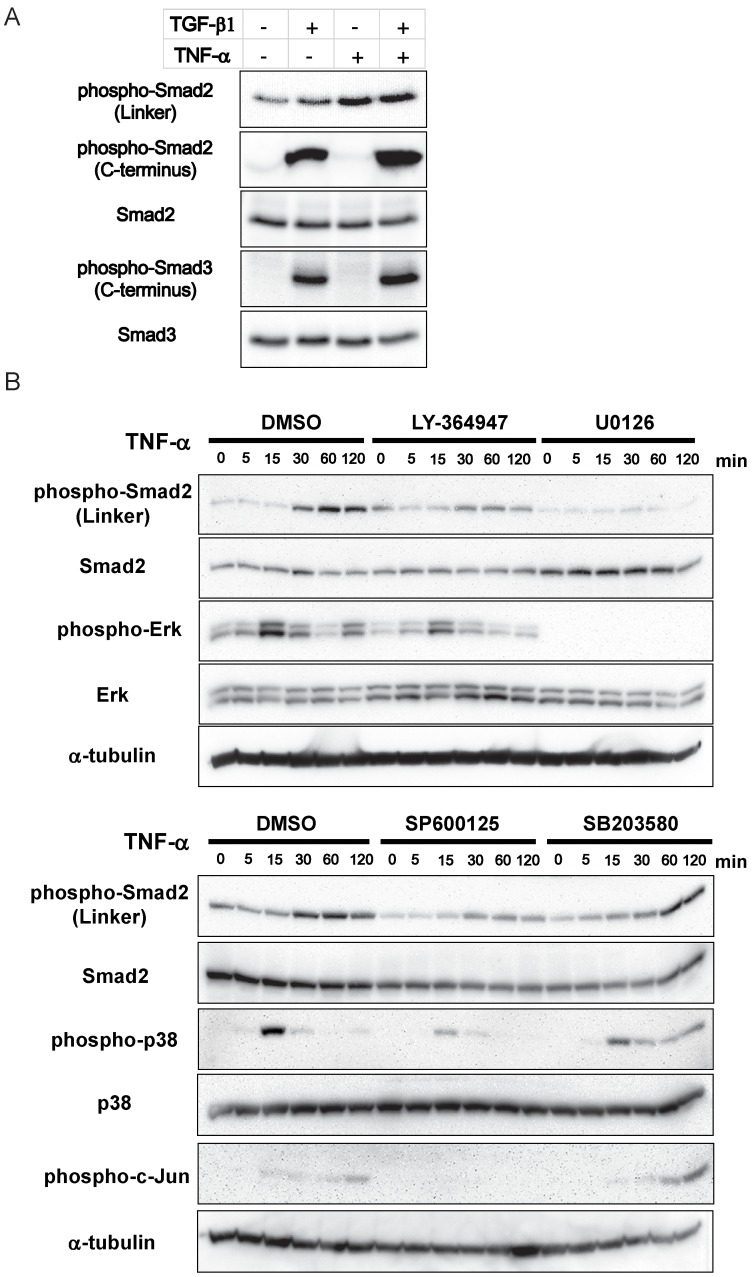
TNF-α phosphorylates Smad2 linker region via MEK-Erk and JNK pathways. (A) A549 cells were stimulated with TGF-β1 and/or TNF-α for 60 min and immunoblotting was performed for total Smad2, phosphorylated Smad2 (linker region: Ser 245/250/255), phosphorylated Smad2 (C-terminal region: Ser 465/467), total Smad3, phosphorylated Smad3 (C-terminal region: Ser 423/425). (B) A549 cells were pretreated with DMSO or chemical inhibitors (LY-364947, U0126, SP600125 and SB203580) for 60 min, followed by TNF-α stimulation. The cell lysates were collected at the indicated time points, and immunoblotting was performed for Smad2, phosphorylated Smad2 (linker region: Ser 245/250/255), Erk, phosphorylated Erk, p38, phosphorylated p38 and phosphorylated c-Jun. α-tubulin was used as loading control.

To explore the potential modulation of Smad signaling by TNF-α, we investigated the phosphorylation of the C-terminal or linker regions of R-Smads. TGF-β strongly elicited phosphorylation of Smad2 and Smad3 C-terminal regions whereas TNF-α did not show any effect. On the other hand, TNF-α stimulation led to phosphorylation of the linker region of Smad2, regardless of TGF-β stimulation (Figure 3A).

Next we further sought to elucidate which kinase is involved in TNF-α-mediated phosphorylation of Smad2 linker region, using chemical inhibitors such as LY-364947, U0126, SP600125 and SB203580 (Figure 3B). TNF-α stimulation led to phosphorylation of Erk, p38 and c-Jun, a substrate of JNK. TNF-α stimulation also elicited phosphorylation of the linker of Smad2 in 30–120 min, reaching a peak at 60 min. LY-364947 failed to abolish this effect, showing the effect of TNF-α independent of TβR-I kinase activity. Of the inhibitors tested, TNF-α-mediated Smad2 linker phosphorylation was abrogated by U0126, a MEK inhibitor which could also abolish the phosphorylation of Erk, a downstream substrate of MEK. The JNK inhibitor, SP600125 abolished phosphorylation of c-Jun, a downstream substrate of JNK, and partially inhibited Smad2 linker phosphorylation. The p38 MAPK inhibitor, SB203580 failed to affect Smad2 linker phosphorylation at the concentration shown to be effective in previous reports.

These results suggested that TNF-α elicits phosphorylation of Smad2 linker region, which might modulate Smad-regulated gene transcription [22]. This effect appeared to be largely mediated by MEK-Erk pathway and probably JNK might also play a role, albeit to a lesser extent (Figure 3B).

### Microarray Analysis Displays Differential Gene Regulation by TGF-β and TNF-α

**Figure 4:**
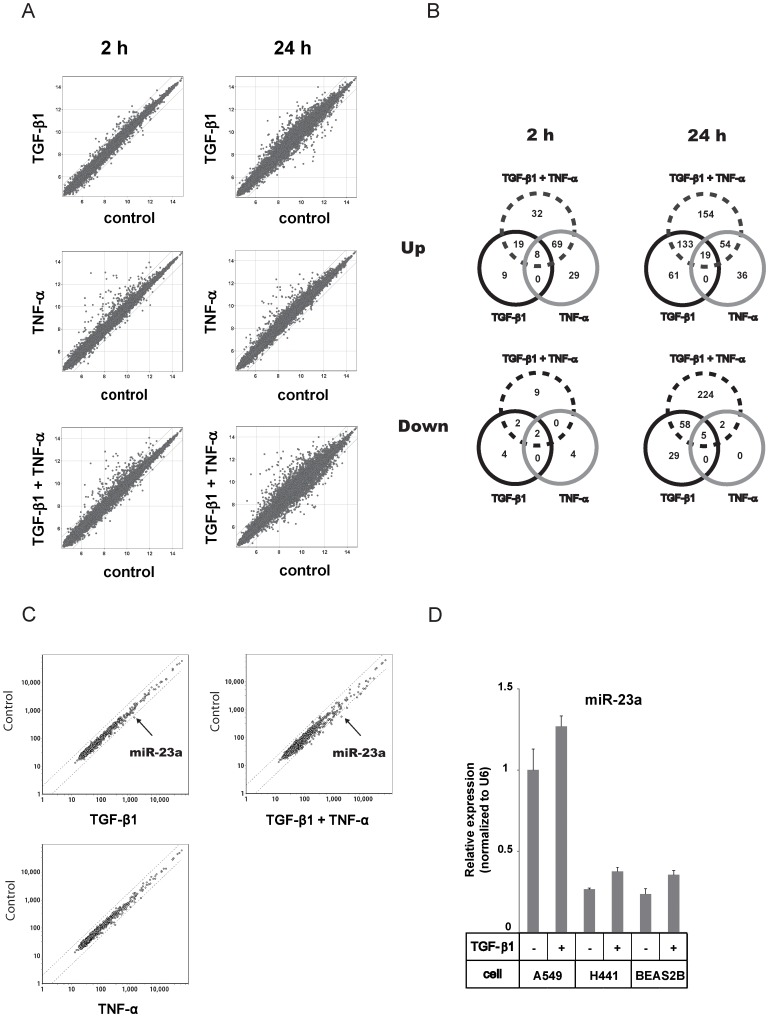
Expression profiling of mRNA and miRNA. (A) Scatter plot representation of the transcripts in the samples treated with TGF-β1 and/or TNF-α for 2 h or 24 h (Y-axis), compared to those of unstimulated control (X-axis). The transcripts are plotted using log2 normalized data. The threshold of transcript levels was set as induced >2.0-fold or repressed <0.5-fold, and is indicated in each scattergram. (B) Venn diagram illustrating the overlap between genes upregulated (Up) or downregulated (Down) by TGF-β1 and/or TNF-α treatment for 2 h or 24 h. The threshold of transcript levels was set as induced >2.0-fold or repressed <0.5-fold. The figures indicate the number of annotated genes. (C) Scatter plot representation of mature miRNAs in the samples treated with TGF-β1 and/or TNF-α for 24 h (X-axis), compared to those of unstimulated control (Y-axis). The threshold of miRNA levels was set as induced >2.0-fold or repressed <0.5-fold, and is indicated in each scattergram. (D) Quantitative RT-PCR for mature miR-23a. A549, H441 and BEAS2B cells were treated with TGF-β1 for 24 h. Expression was normalized to that of U6. Error bars: SD.

To obtain comprehensive insights into the transcriptional changes occurring upon TGF-β and/or TNF-α treatment, gene expression profiling was performed. Total RNA samples were prepared from A549 cells treated with TGF-β and/or TNF-α for 2 h or 24 h, and were further analysed by microarray analysis (Figure 4A). The transcripts induced >1.5-fold or repressed <0.67-fold, including those not annotated, were listed in File S1 and File S2 (the data at 2 h and 24 h, respectively).

For the further analysis, we set the threshold of transcript levels as induced >2.0-fold or repressed <0.5-fold, and excluded unannotated transcripts. Thus we identified genes with altered expression as compared to unstimulated control, in 3 comparative sets, i.e. TGF-β-stimulated, TNF-α-stimulated and TGF-β/TNF-α-stimulated groups (Figure 4B).

As an overall tendency, TGF-β-regulated and TNF-α-regulated genes were largely exclusive, displaying differential regulation of gene transcription. In addition, upregulated genes were identified much more than those downregulated. The genes induced by TNF-α were more prominent than TGF-β at 2 h, whereas the number of genes upregulated by TGF-β was much greater at 24 h as compared to those at 2 h, or those by TNF-α. At 24 h, there was a fraction of genes which were up- or downregulated by TGF-β and TNF-α costimulation, but not either of them.

Next we employed the published data sets of microarray, which were performed in A549 cells stimulated with TGF-β [23], [24]. Considering induction >2.0-fold as significant, we extracted potential TGF-β target genes commonly induced in three independent studies, i.e. 17 genes at 2h, and 129 genes at 24 h, which are shown in File S3.

### Subsets of Genes Differentially or Cooperatively Regulated by TGF-β and TNF-α

Several transcription factors have been implicated in the transcriptional repression of E-cadherin, including *SNAI1* (Snail), *SNAI2* (Slug), *ZEB1*, *ZEB2* and *TWIST1* [25], [26]. Of these, *SNAI1* was rapidly induced by TGF-β at 2 h whereas TNF-α rather suppressed it, implying the differential mechanisms to regulate EMT. At 24 h, E-cadherin (*CDH1*) was clearly downregulated by TGF-β (0.27-fold) while the suppressive effect of TNF-α was modest (0.78-fold). In accordance, TGF-β and TNF-α upregulated the expression of N-cadherin (*CDH2*) up to 2.32-fold and 1.47-fold, respectively. The synergic effect of TGF-β and TNF-α was also observed with regard to transcriptional regulation of *CDH1* and *CDH2*, consistent with the results in Figure 1.

Furthermore, the genes upregulated more than 3.0-fold at 2 h or 24 h, were subclassified and listed in Table 1 and 2, according to the known functions. As an acute response at 2 h, TNF-α potently induced a number of cytokines/chemokines such as *CCL2* (MCP-1), *CCL5* (RANTES), *CCL20*, *CXCL1*, *CXCL8* (IL8), *IL1A*, *IL1B* and *IL6*. Signaling components of TNF-α-NFκB pathway were also upregulated including *TNF*, *TRAF1*, *NFKB1*, *NFKBIA* and *NFKBIE*. Furthermore, cell adhesion molecules such as *VCAM1* and *ICAM1* were induced. At 2 h after stimulation, TGF-β induced limited number of genes including the known direct targets such as *SMAD7* and *HEY1*. At 24 h, TGF-β stimulation led to enhanced expression of various genes categorized as (i) regulator of small GTPase, (ii) cell adhesion molecule, and (iii) ECM, whereas TNF-α stimulation only showed minor effects on them.

**Table 1:** Upregulated genes by TGF-β1 and/or TNF-α, 2 h after stimulation.

2 h	TGF-β1		TNF-α			TGF-β1+ TNF-α	
**Growth factor and receptor**	BMBI			INHBA		INHBA	
						HBEGF	
**Cytokine and chemokine**	IL11			CCL2	IL1A	CCL2	IL1A
				CCL5	IL6	CCL5	IL6
				CCL20	IL8	CCL20	IL8
				CXCL1	CSF2	CXCL1	CSF2
				CXCL2	TNF		TNF
				CXCL3	IL1B		
					IL23A		
**Protease and inhibitor**				SERPINA3		SERPINA3	
**Regulator of small GTPase**	ARL4D			RND1		RND1	
	RND1					GEM	
						RRAD	
**Cell adhesion**				ICAM1		ICAM1	
				VCAM1			
**Others**	SMAD7			TNFAIP2	TNFRSF9	TNFAIP2	TNFRSF9
	HEY1			TNFAIP3	TRAF1	TNFAIP3	TRAF1
	ANGPTL4			TNFAIP6	NFKBIA	TNFAIP6	NFKBIA
				SOD2	NFKBIE	SOD2	NFKBIE
				STAT5A		STAT5A	NFKB1
						VDR	

**Table 2:** Upregulated genes by TGF-β and/or TNF-α, 24 h after stimulation.

24 h	TGF-β1		TNF-α		TGF-β1+ TNF-α	
**Growth factor and receptor**	CTGF		IGFBP1		INHBA	TGFBR1
					PDGFB	PDGFRA
					BMP2	HBEGF
					FGF5	CTGF
**Cytokine and chemokine**	IL11		CCL2	IL1A	CCL2	IL1A
			CCL5	IL6	CCL5	IL6
			CCL20	IL8	CCL20	IL8
			CXCL1	EBI3 (IL27)		IL11
						IL32
						IL33
**Protease and inhibitor**	MMP2		MMP1		MMP1	SERPINA3
	ADAM19		SERPINA3		MMP2	SERPINB8
	SERPINE1		SERPINB8		MMP9	SERPINE1
					PLAT	SERPINE2
					PLAU	ADAM19
**Regulator of small GTPase**	RASGRP1	RHOU			RASGRP1	RHOU
	RASGRP3	DOCK2			RASGRP3	DOCK4
	RASGRF2	DOCK4			RASGRF2	RND1
**Cell adhesion**	ITGA11	CDH4	ICAM1		ITGA2	CDH4
	ITGB6	CDH19			ITGA5	CDH19
		ESAM			ITGB3	ICAM1
						DSC2
**Extracellular matrix**	COL4A1	PODXL	LAMC2		COL4A1	SPOCK1
	LAMC2	HAPLN3			LAMB3	PODXL
	SPOCK1				LAMC2	HAPLN3
**Others**	TNFAIP6	ANGPTL4	TNFAIP3	VDR	TNFAIP3	ANGPTL4
	LOX	ABLIM3	TNFRSF9		TNFAIP6	ABLIM3
		SLN	NFKB1		TNFRSF9	SLN
		TAGLN	NFKB2		NFKB1	VDR
		MYOCD			NFKB2	FERMT1

Regulators of small GTPase included guanine nucleotide exchange factors (GEFs) such as *RASGRP1*, *RASGRP3*, *RASGRF2*, *DOCK2* and *DOCK4*, which activate Ras, Rac and Rap1, as well as the members of Rho family GTPases such as *RND1* and *RHOU*. Cell motility and morphological changes are regulated by small GTPases such as Ras, Rac, Cdc42 and Rho families, which can be modulated downstream of TGF-β signaling [27]. Cell adhesion molecules included the members of cadherin such as *CDH4* and *CDH19*, as well as those of integrin family such as *ITGA2*, *ITGA5*, *ITGA11*, *ITGB3* and *ITGB6*. TGF-β stimulation further resulted in highly enhanced expression of the components of ECM such as collagen (*COL1A1*, *COL4A1*, *COL4A2*, *COL5A1*, and *COL5A2*) and laminin (*LAMC2*). As integrins function as receptors for ECM, these expression changes were speculated to modulate bidirectional cell signaling and cellular phenotype [28].

There were two findings regarding the cooperative effects of TGF-β and TNF-α. In the group costimulated with TGF-β and TNF-α, growth factors and their receptors were upregulated including *BMP2*, *INHBA*, *HBEGF*, *FGF5*, *CTGF*, *PDGFB*, *PDGFRA* and *TGFBR1*. Moreover, the expression of proteases and their inhibitors were enhanced, such as MMPs, plasminogen activators (*PLAT* and *PLAU*), and protease inhibitors called as serpins (*SERPINA3*, *SERPINB8*, *SERPINE1* and *SERPINE2*). These proteases and protease inhibitors participate in remodeling of ECM as well as activation/processing of cytokines or growth factors. Thus TGF-β and TNF-α might cooperatively alter the extracellular milieu surrounding cancer cells structurally as well as functionally. TNF-α and TGF-β also upregulated genes involved in hyaluronan remodeling, such as *HAS2*, *HAS3* and *HAPLN3*, which might have a role in EMT regulation through hyarulonan-CD44 interaction [29]. Strong induction of *PODXL* (podocalyxin) following TGF-β treatment was also observed as reported previously [30].

### MicroRNA Array Analysis Reveals miR-23a as a Target of TGF-β

Noncoding microRNAs (miRNAs) are also critical components of cellular signaling implicated in the regulation of EMT. In the list of microarray data, we noted distinct populations of genes which were suppressed by TGF-β and TNF-α, and we hypothesized that their expression could be regulated by miRNAs. In search of miRNAs induced by TGF-β and TNF-α, we performed miRNA array analysis (Figure 4C). The normalized data of TGF-β-stimulated, TNF-α-stimulated and TGF-β/TNF-α-stimulated groups were listed in File S4. Mature miRNAs induced >2.0-fold included miR-23a in the TGF-β-stimulated sample (2.61-fold), while any miRNA with >2.0-fold induction was not noted in the TNF-α-stimulated sample. Costimulation with TGF-β and TNF-α induced miR-23a (3.32-fold), miR-720, miR-4275 and miR-4285.

Next we validated the induction of miR-23a by TGF-β in lung cancer/epithelial cell lines, i.e. A549 and H441 lung cancer cells as well as BEAS2B transformed bronchial epithelial cells. In all these cell lines, TGF-β could induce mature miR-23a as confirmed by quantitative RT-PCR (Figure 4D).

### Integrated Analysis of mRNA and miRNA Arrays Reveals Potential Targets Regulated by TGF-β and miR-23a

We proceeded to explore the potential participation of miR-23a in the regulation of TGF-β-elicited EMT, and searched for target genes which could be regulated by miR-23a.

It has been demonstrated that miR-23a promotes invasive capacity of colon cancer cells [31], and miR-23a targets E-cadherin to modulated EMT in lung cancer cells [32]. However, miR-23a inhibition could not modulate TGF-β-elicited EMT in A549 cells (Figure S1). Additionally, transfection of synthetic precursor miR-23a (pre-miR-23a) failed to alter the expression of E-cadherin or N-cadherin (Figure S1). Thus miR-23a did not appear to play a role in our model of EMT.

**Figure 5:**
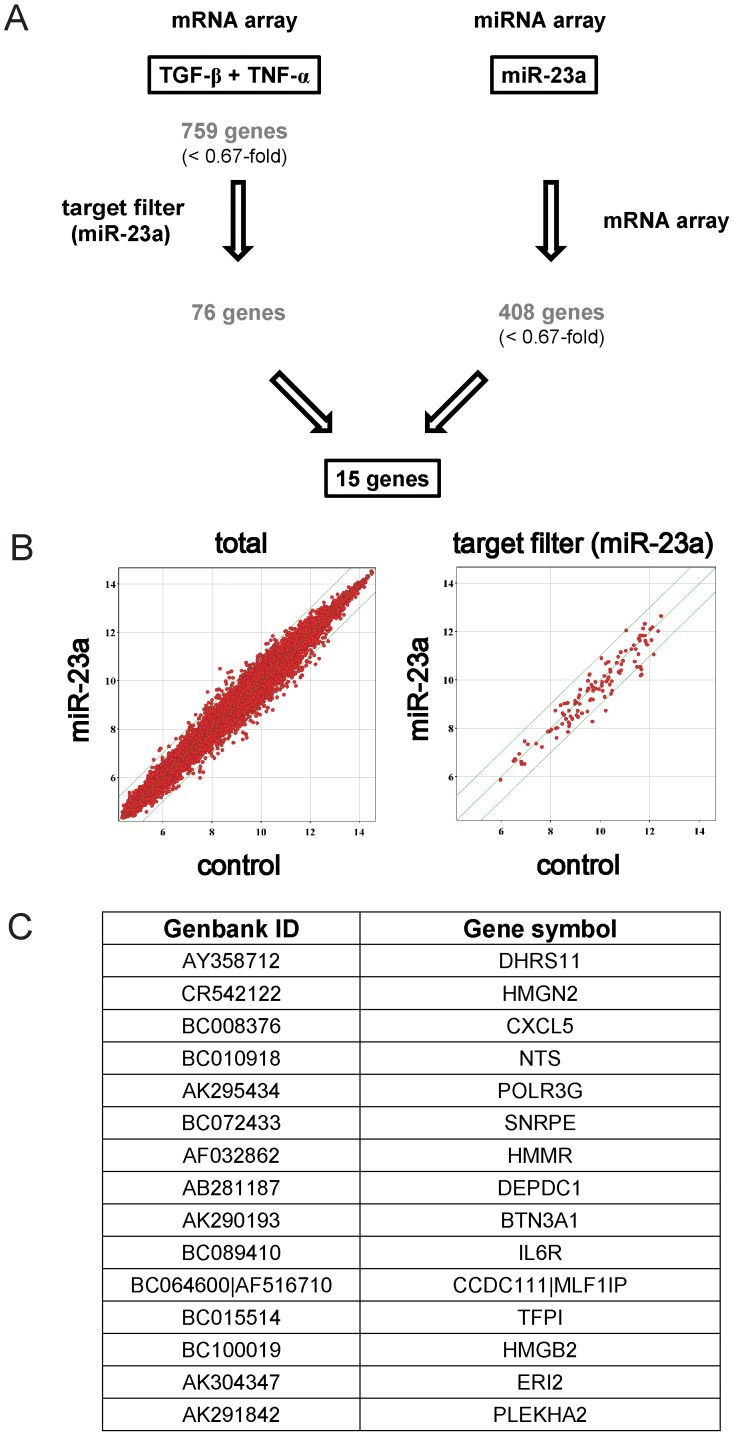
Putative targets of TGF-β-induced miR-23a. (A) Schematic description of integrated analysis of mRNA and miRNA arrays. Gene expression signature in the cells treated with TGF-β1 and TNF-α revealed 759 annotated genes downregulated <0.67-fold. Out of them, the target filter program listed 76 genes as putative miR-23a targets. Transfection of synthetic pre-miR-23a in A549 cells identified 408 genes downregulated <0.67-fold. There were 15 genes shared in these two groups. (B) Scatter plot representation of the transcripts in A549 cells transfected with control (X-axis) or synthetic pre-miR-23a (Y-axis) for 48 h. The transcripts are plotted using log2 normalized data. The threshold of transcript levels was set as induced >2.0-fold or repressed <0.5-fold, and is indicated in each scattergram. Plots of total transcripts (right), and those selected by the target filter program (right), are presented. (C) The list of 15 genes identified by integrated analyses.

Next we explored to identify potential miR-23a targets. Utilizing the miRNA target filter in IPA analysis, 76 genes were extracted out of 759 genes which were downregulated <0.67-fold by TGF-β and TNF-α (Figure 5A). To further validate target genes potentially regulated by miR-23a, we performed gene expression profiling in the cells transfected with pre-miR-23a or negative control, which yielded 408 genes downregulated <0.67-fold by pre-miR-23a transfection (Figure 5B). Combining these array results, 15 genes were extracted as targets of TGF-β/TNF-α as well as miR-23a (Figure 5A and 5C).

**Figure 6:**
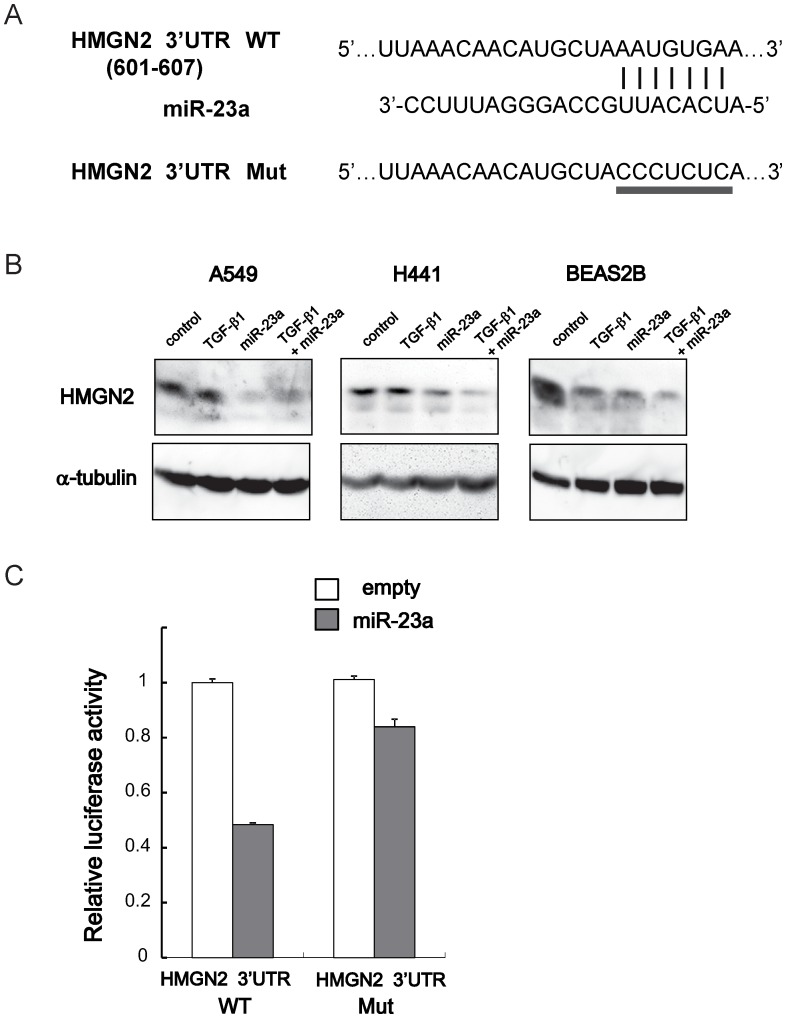
HMGN2 is regulated by miR-23a. (A) Putative miR-23a binding sequence in the 3′UTR of human HMGN2 mRNA (WT: wild-type). The underlined sequence shows the nucleotides generated by mutagenesis to abolish the binding of miR-23a (Mut: mutant). (B) Immunoblotting for HMGN2 in A549, H441 and BEAS2B cells transfected with control or synthetic pre-miR-23a for 48 h, followed by TGF-β1 stimulation for additional 48 h. α-tubulin was used as loading control. (C) HEK293T cells were transfected with luciferase reporters containing the HMGN2 3′UTR with wild-type or mutated target site as shown in Fig. 6A, along with empty or pri-miR-23a expression vector. Luciferase assay was performed 48 h after transfection.

Out of the 15 genes, we selected *HMGN2* (high motility group nucleosomal 2, also known as *HMG-17*) for further analyses, since it is implicated in cellular differentiation and cancer [33], [34]. The putative miR-23a binding sequence in the 3′UTR of human HMGN2 transcript was identified by Targetscan (Figure 6A). To confirm miR-23a-mediated suppression of HMGN2, immunoblotting for HMGN2 was performed after TGF-β stimulation and/or pre-miR-23a transfection (Figure 6B). TGF-β downregulated HMGN2 in BEAS2B cells whereas it did not affect HMGN2 expression in A549 or H441 cells at protein level. On the other hand, pre-miR-23a transfection clearly led to suppression of HMGN2 in all these cell lines.

To investigate the direct regulation of HMGN2 by miR-23a, wild-type 3′UTR of HMGN2 mRNA was subcloned downstream of the luciferase reporter. We also constructed the reporter vector with mutations in the putative miR-23a binding site (Figure 6A). As expected, luciferase activity was markedly reduced when the miR-23a expression vector was cotransfected, whereas the miR-23a-induced decrease in luciferase activity was abolished for the mutant reporter vector (Figure 6C).

## Discussion

The cooperative action of TGF-β and TNF-α on EMT attracts increasing attention since it reflects the cancer microenvironment infiltrated with inflammatory cells, and provides a model where inflammatory signals enhance tumor progression [13], [14], [15]. This idea is also supported by the observation that activated macrophages can enhance TGF-β-mediated EMT [35], [36]. Recently it has been also reported that tumor cells treated with TGF-β and TNF-α generates a population of stem cells, further evoking attention on their possible crosstalks [37].

The present study demonstrated that TGF-β and TNF-α synergically induces EMT in A549 lung cancer cells. Their effect was mostly dependent on Smad pathway, and TNF-α could induce phosphorylation of Smad2 linker region, suggesting the possible modulation of Smad-regulated gene transcription. Gene expression signature revealed cohorts of genes differentially or cooperatively regulated by TGF-β and TNF-α. Furthermore, miRNA array analysis identified miR-23a, as a target of TGF-β. Integrated analysis of mRNA and miRNA expression profiles, combined with in silico screening, provided a list of genes, possibly regulated via miR-23a. We further validated miR-23a-mediated regulation of HMGN2.

The miR-23a is transcribed from the miR-23a∼27a∼24-2 cluster on chromosome 19p13, followed by cleavage to yield mature miR-23a. The miR-23b∼27b∼24-1 cluster is its paralog on chromosome 9q22, and mature miR-23a differs by just one nucleotide compared to miR-24, suggesting their overlapping targets [38]. These three miRNAs of this cluster are derived from a single primary transcript, but the levels of each can vary because of post-transcriptional processing [38], [39].

Upregulation of miR-23a has been documented in a variety of human cancers including gastric cancer, glioblastoma, breast cancer, and pancreatic cancer [40]. Consistent with our observation, it has been shown that miR-23a or miR-24 can be induced by TGF-β in keratinocytes and hepatocellular carcinoma cells [41], [42]. Furthermore, upregulation of miR-23a in association with TGF-β signaling has been reported in non-small cell lung cancer cells [32]. In this study, we could not find any effect of miR-23a on EMT markers, though it does not exclude the possibility that miR-23a might have an impact on molecular events accompanying EMT. Further studies are warranted to clarify the exact role of miR-23a in lung cancer cells undergoing EMT.

Out of 15 genes chosen as putative miR-23a targets by integrated analyses, IL-6 receptor (*IL6R*) has been experimentally validated [43]. In addition to this previous report, we have for the first time, validated HMGN2 as a direct target of miR-23a, in the current study. HMGN2 regulates transcription via alteration of chromatin structure through interfering with the binding of linker histone H1 to the nucleosome. Recently, microarray analyses revealed genes potentially regulated by HMGN2 in A549 cells, which suggested HMGN2-mediated modulation of diverse cell signals [44]. Thus HMGN2 has been implicated as a downstream effector of miR-23a.

In conclusion, integrated analyses of gene and miRNA expression profiling delineated cooperative and differential action of TGF-β and TNF-α, and unraveled potential roles of miRNAs. We have shown that miR-23a was induced by TGF-β, and identified HMGN2 as a target of miR-23a. Our findings shed light on the complexity of molecular events accompanying EMT, elicited by TGF-β and TNF-α.

## Supporting Information

Figure S1 **Immunoblotting for E-cadherin and N-cadherin in A549 cells transfected with control versus synthetic pre-miR-23a, or control versus miR-23a inhibitor for 48 h, followed by TGF-β1 stimulation for additional 48 h.** α-tubulin was used as loading control.
(EPS)

Table S1 **Primers used for quantitative RT-PCR in this study.**
(EPS)

Table S2 Primers used for the construction of 3′UTR reporter vector and pri-miRNA expression vector.
(EPS)

File S1 **The transcripts at 2 h induced >1.5-fold or repressed <0.67-fold, including those not annotated.**
(XLSX)

File S2 **The transcripts at 24 h induced >1.5-fold or repressed <0.67-fold, including those not annotated.**
(XLSX)

File S3 **Integrated analysis with the published data sets of microarray, which were performed in A549 cells stimulated with TGF-β.** Considering induction >2.0-fold as significant, we extracted potential TGF-β target genes commonly induced in three independent studies, i.e. 17 genes at 2 h, and 129 genes at 24 h.
(XLSX)

File S4 **MicroRNA array analysis.** The normalized data of TGF-β-stimulated, TNF-α-stimulated and TGF-β/TNF-α-stimulated groups.
(XLSX)
