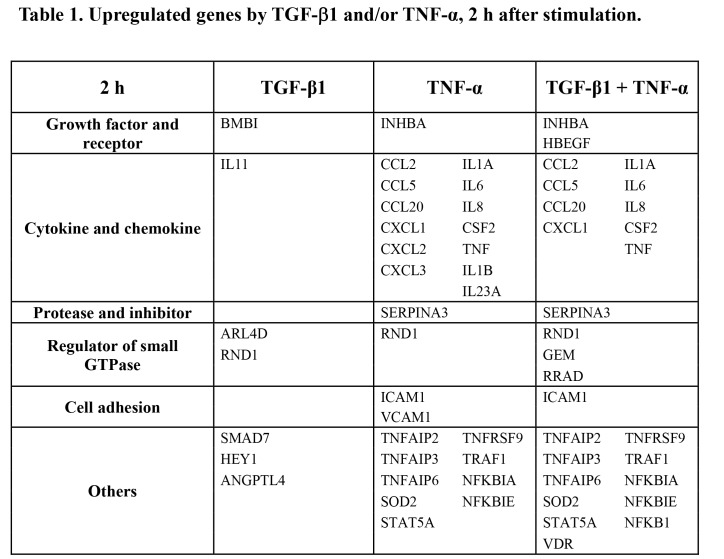# Correction: An Integrated Expression Profiling Reveals Target Genes of TGF-β and TNF-α Possibly Mediated by MicroRNAs in Lung Cancer Cells

**DOI:** 10.1371/annotation/874e0b87-0383-45ec-a250-3a4cd087dc86

**Published:** 2014-01-14

**Authors:** Akira Saito, Hiroshi I. Suzuki, Masafumi Horie, Mitsuhiro Ohshima, Yasuyuki Morishita, Yoshimitsu Abiko, Takahide Nagase

SERPINA3 is incorrectly under "Protease and inhibitor." It should correctly be under the column "TNF-α." Please see the corrected Table 1: 

**Figure:**